# Trends in dermatology consultations in the COVID‐19 era in Cameroon

**DOI:** 10.1002/ski2.113

**Published:** 2022-04-01

**Authors:** Edgar Mandeng Ma Linwa, Odette Berline Sigha, Angelique Jacquie Djeumen Touka, Charlotte Eposse Ekoube, Esther Eleonore Ngo Linwa, Michael Ngenge Budzi, Martin Geh Meh, Henry Fomukong Nzozone, Emmanuel Armand Kouotou, Anne‐Cecile Zoung Kanyi Bissek

**Affiliations:** ^1^ Faculty of Health Sciences University of Buea Buea Cameroon; ^2^ Hopital Laquintinie de Douala Douala Cameroon; ^3^ Faculty of Health Sciences University of Bamenda Bamenda Cameroon; ^4^ Faculté de Médecine et des Sciences Pharmaceutiques Université de Douala Douala Cameroun; ^5^ Non‐Communicable Disease Prevention and Control Programme Cameroon Baptist Convention, Health Services Yaounde Cameroon; ^6^ Faculté de Médecine et des Sciences Biomédicales de Yaoundé Yaoundé Cameroon

## Abstract

**Introduction:**

With the advent of COVID‐19, a highly infectious viral pandemic, first declared in Cameroon in March 2020, access to expert dermatology care was postulated to decrease. We carried out a descriptive study to understand the epidemiology of skin diseases amongst patients consulting at an outpatient dermatology clinic and its variations with the onset of COVID‐19.

**Methods:**

We conducted a retrospective database study over a period of 2 years at Laquintinie hospital, Cameroon. Two periods were distinguished: 1 March 2019 to 29 February 2020 (pre‐COVID‐19 era) and 1 March 2020 to 28 February 2021 (COVID‐19 era).

**Results:**

Overall, 5946 patients with one or more clinical conditions were analysed. The most common age group described was young adults (18–40 years). Females consulted twice as much as males (M/F ratio; 1:1.7). The mean age was 28.9 (±18.0) years with an age range of 1 month to 89 years. Infectious dermatoses predominated. However, the single most prevalent dermatologic condition was acne. There was a 35.6% drop in consultation frequency in the COVID‐19 era.

**Conclusion:**

Three in four patients consulting at dermatology clinic are below 40 years, with a female predominance. Epidemiological profile of dermatoses in Cameroon is similar to that in the rest of Africa. Consultation frequency decreased in the COVID‐19 era but trends in consultations were rather unaltered except for patients above 50 years where eczema and scabies increased. More studies should be conducted to understand these trends better at a national level and envisage training of health personnel on prevalent dermatoses.

## Graphical abstract

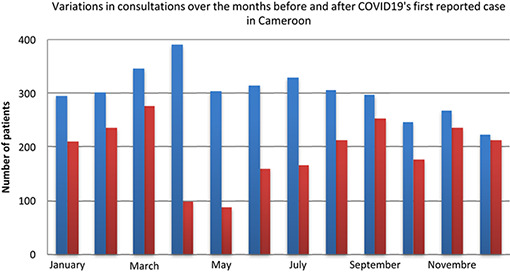

1

**What is already known about this topic?**

- The most prevalent skin conditions in developing countries remain infectious while those in developed countries are malignant conditions.
- Most patients consulting in developing world are youths as opposed to older patients in developed countries.
- Access to dermatology services has been reduced due to COVID‐19 as manifested by lower frequency of dermatology consultations.

**What does this study add?**

- Overall, infectious dermatoses (30.3%) predominate in outpatient consultations. However, the single most prevalent dermatology condition is acne (14.3%) closely followed by eczema (12.8%).
- Three in four patients consulting at dermatology clinic are below 40 years, with a female predominance. Dermatology in Cameroon can be considered to have a youthful patient population.
- Consultation frequency decreased (by 35.6%) in the COVID‐19 era but trends in consultations were rather unaltered except for scabies and pityriasis rosea which increased and decreased, respectively. In patients above 50 years (patients at risk for COVID‐19), consultation frequency for eczema and scabies increased. More studies should be conducted to understand these trends better at a national level.

## 2 INTRODUCTION

Despite the high burden of skin diseases,^1^, ^2^, ^3^ and the documented morbidity in Africa, access to expert dermatology care is limited. Africa still has a great deficiency in dermatologists with fewer than one dermatologist per one million people.^4^ Furthermore, there's a low amount of dermatology research carried out in sub‐Saharan Africa.^5^ The most recent study that described the epidemiological profile of patients consulting at a dermatology clinic was carried out by Bissek et al. in 2002. They found that the most frequent reason for consultation was pruritus (46.0%). The five main diagnoses were allergic reactions (34.0%); infections (20.0%); skin appendage disorders (15.0%) and pigmentation disorders (4.0%). Acne, pruritus and pigment disorders were commoner chief complaints in women than in men. Conversely, scars and ulcerations were commoner chief complaints in men than in women.^6^

Epidemiological description of dermatoses is pivotal for understanding the patterns and limiting the burden and morbidity of skin diseases. With the onset of COVID‐19, a highly infectious viral pandemic, first declared in Cameroon in 2 March 2020,^7^ access to expert dermatology care was postulated to decrease.^8^ With limitations of movements and gatherings, in addition to lockdowns during the pandemic, it would be expected that patients who still seek consultation will do so for pathologies that are pressing, urgent and highly distressing. Knowledge of the most prevalent skin conditions can help guide training sessions of other health professionals in order to disseminate dermatology care. Knowledge of prevalent dermatoses during the COVID‐19 era can help recognise pathologies that are urgent and distressing. We, therefore, carried out a descriptive study to understand the epidemiology of skin diseases amongst patients consulting at the outpatient dermatology clinic in a tertiary hospital and its variations with the onset of COVID‐19.

## 3 METHODS

The site selected for this study was Laquintinie Hospital Douala. This is a hospital that serves the population of the economic capital of Cameroon, Douala. About a third of the population of Douala lives in poverty and a third of the poor prefer informal facilities for their health care.^9^ Laquintinie is located in Douala I subdivision and receives averagely 150 000 patients per year.^10^ Dermatology consultations constitute less than 5% of overall hospital consultations yearly (unpublished hospital statistics). The hospital has two dermatologists who conduct outpatient consultations every day. However, in some units such as paediatric unit, dermatology cases are sometimes consulted and referrals made to dermatologists only if cases are non‐responsive to treatment. We conducted a retrospective database study over a period of 2 years: 1 March 2019– 31 March 2021. Two periods were distinguished: 1 March 2019–29 February 2020 (pre‐COVID‐19 era) and 1 March 2020–28 of February 2021 (COVID‐19 era). Data collection was done using consultation registers. These registers were conceived to record hospital consultation statistics and as such detailed clinical data were not documented. It records the name of the patient, the date, age, sex, residence, profession, presumptive diagnosis and labs done. For the purpose of the study, only age, sex, occupation, professions, residence and presumptive diagnoses were collected from registers in an anonymous way. Clinical diagnoses as reported by dermatologists were classified according to the WHO International Classification of Diseases 11th Edition (ICD‐11).^11^ For patients with multiple diagnoses, all clinical conditions were recorded. As such the number of diagnoses do not coincide with the number of patients as some patients had more than one diagnosis. Data were represented as counts and frequencies and displayed in tables. Sampling was consecutive and exhaustive; all available relevant data (demographic data and clinical condition(s) diagnosed at consultation) were included and analysed. Patients with incomplete data were excluded from the study. Follow‐up visits were excluded from the analysis. Administrative clearance was obtained from the Director of the Laquintinie Hospital Douala. Ethical clearance was not requested since this research involves existing hospital data, codified and anonymised. Furthermore, the current study is part of the hospital's strategic research plan. Data were anonymised and ethical considerations were respected. SPSS version 20 was used for statistical analyses. Categorical variables were compared using the Chi square test. Binomial regression analysis was used to find independent associations between variables and outcome. Odds ratios were used to express risk and the 95% Confidence Interval was displayed. Significance level was set at *p* < 0.05.

## 4 RESULTS

Overall, 5946 cases were analysed. Females consulted almost twice as much as males (M/F ratio; 1:1.7). The mean age was 28.9 years (±18.0) with an age range of 1 month to 89 years. The most common age group consulting at the outpatient dermatology clinic of Laquintinie Hospital was the young adults group aged between 18 and 40 years (49.2%, *n* = 2925), and they constituted almost half of all consultations. The paediatric population aged 0–17 years constituted 26.2% (*n* = 1559) of the population. The elderly population aged >50 years constituted 13.5% of the population (*n* = 800). Most patients who consulted were students (4/10). Most patients (34.2%, *n* = 2090) were living in Douala III subdivision, 14.8% (*n* = 845) in Douala II, 12.4% (*n* = 759) in Douala I, 10.1% (*n* = 620) in Douala IV, 9.6% (*n* = 599) in Douala V, while only 5.7% (*n* = 349) were living out of Douala. Only three patients came from Douala VI. The most common skin disorders were infectious diseases (30.3%). This was closely followed by dermatitis and eczematous conditions (19.4%), acne and acneiform disorders (14.3%), papulosquamous disorders (11.7%) and skin tumours (6.3%) as depicted in Table 1. In the pre‐COVID‐19 era, dermatology consultations constituted 4.3% of overall consultations (3618/83 800). In the COVID‐19 era, this proportion went down to 3.2% (2328/72 395) [OR = 1.554 (1.504–1.606) 95% CI, *p* < 0.001]. There was a 13.6% drop in overall consultation in the hospital in the COVID‐19 era (11 405/83 800). Dermatology consultations contributed to 11.3% of this drop (1290/11 405).

**TABLE 1:** Distribution of grouped dermatological diseases amongst different age groups

Diagnosis (International Classification of Diseases 11th Edition: ICD0‐11 CODES)	Age (years				Number (%)
	0–17 years	18–40 years	41–50 years	51 years and older	
**Infectious dermatoses**	**540**	**848**	**192**	**225**	***N* = 1805 (30.3)**
Fungal infections (EA60.Z)	147	371	90	109	717
Bacterial infections (1B21.2Z. 1C44. 1B7Y)	161	159	43	39	402
Scabies (1G04)	126	230	42	55	453
Viral infections (EA3Z, EH10, 1F0Z,1A95 1E76, 1E80)	123	113	19	26	281
Filaria, and larva migrans (1F66.Z, 1F68.2)	4	6	2	4	16
**Dermatitis and eczematous disorders**	**311**	**490**	**144**	**208**	***N* = 1153 (19.4)**
Eczema (EA80)	194	325	97	144	760
Lichen simplex (EA83)	25	103	35	36	199
Erythroderma (EB10)	6	7	1	10	24
Xerosis cutis and ictyosis (ED54, EC20.Z)	7	20	6	14	47
Others (EA81.Z, EA86.0)	79	35	6	7	127
**Acne and acneiform disorders** (ED80.Z)	**83**	**701**	**48**	**16**	***N* = 848 (14.3)**
**Papulosquamous disorders**	**379**	**215**	**48**	**54**	***N* = 696 (11.7)**
Pityriasis rosea (EA10)	57	94	10	4	161
Psoriasis (EA90.Z)	2	25	9	27	63
Prurigo (EC91.Z)	321	97	29	23	470
**Skin tumours** EE60, EF02.1, 2E80.00, EK70.Z, 2F21.0, 1A95, 1E76, 1E80, 2F22, LC00, 2F21.0, 2C3Z)	**55**	**196**	**46**	**63**	***N* = 360 (6.1)**
**Vascular disorders**	**59**	**145**	**35**	**34**	***N* = 273 (4.6)**
Urticaria (EB05)	29	125	24	18	196
Lymphoedema (BD93.Z)	0	3	6	10	19
Others (2E81.2Y, 2F23.0, EA86.0, BD74.1Z, BD74.2)	30	17	5	6	58
**Pigmentary disorders** (ED63.0, ED63.Y, ED63.Z)	**50**	**104**	**53**	**49**	***N* = 256 (4.3)**
**Disorders of keratinisation** (ED5Y)	10	16	9	5	***N* = 40 (0.7)**
**Disorders of skin appendage** (Alopecia [ED70.Z])	**5**	**27**	**12**	**5**	***N* = 49 (0.8)**
**Idiopathic pruritus/psychoneurosis** (EC90.4, EC90.Y)	4	37	9	27	***N* = 77 (1.3)**
**Ulcerative disorders** (ME60.Z)	2	6	11	6	***N* = 24 (0.4)**

*Note*: A *p*‐value <0.05 was considered significant.

Acne was the most common single diagnosis. More than 1 in 10 patients (14.3%) consulting had acne. Females predominated in all skin conditions except, prurigo, scabies and lichen simplex where their male counterparts were predominant (Table 2). Significant increase in frequency were noted for scabies (+3.8%) [OR = 1.617 (1.355–1.930) 95% CI, *p* < 0.001] and lichen simplex (+1.6%) [OR = 1.570 (1.195–2.062) 95% CI, *p* < 0.001] as shown in Table 3. However, there was a drop in consultation frequency for pityriasis rosea (−0.9%) [OR = 0.715 (0.517–0.989) 95% CI, *p* = 0.043]. On multiple regression analysis, scabies [OR = 1.640 (1.235–2.177) 95% CI, *p* = 0.001] and pityriasis rosea [OR = 1.697 (1.400–2.057) 95% CI, *p* < 0.001] were significantly increased and decreased respectively in the COVID‐19 era as shown in Table 4.

**TABLE 2:** Gender differences in top 10 single diagnoses at outpatient dermatology consultations

Skin disease (ICD‐11 CODE)	Male	Female	Total
Acne (ED80.Z)	205 (9.3%)	643 (17.2%)	848 (14.3%)
Eczema (EA80)	277 (12.6%)	482 (12.9%)	760 (12.8%)
Prurigo (EC91.Z)	197 (8.9%)	273 (7.3%)	470 (7.9%)
Scabies (1G04)	188 (8.5%)	264 (7.1%)	453 (7.6%)
Dermatophyte infections (1F28.Z)	96 (4.4%)	158 (4.2%)	254 (4.3%)
Lichen simplex (EA83)	83 (3.8%)	116 (3.1%)	199 (3.3%)
Urticaria (EB05)	53 (2.4%)	143 (3.8%)	196 (3.3%)
Pityriasis versicolour (1F2D.0)	78 (4.4%)	112 (3.0%)	190 (3.2%)
Pityriasis rosea (EA10)	45 (2.0%)	120 (3.2%)	165 (2.8%)
Folliculitis/pseudofolliculitis (ED9Y)	81 (3.7%)	73 (2.0%)	154 (2.6%)

**TABLE 3:** Pattern of change of commonest dermatological diseases in the COVID‐19 era

Skin disease (ICD‐11 CODE)	Pre‐COVID‐19 era (*n* = 3618)	COVID‐19 era (*n* = 2328)	Total	OR (95% CI)	*p*‐value
Acne (ED80.Z)	498 (13.8%)	350 (15.0%)	848 (14.3%)	1.092 (0.963–1.239)	0.172
Eczema (EA80)	468 (12.9%)	292 (12.5%)	760 (12.8%)	0.970 (0.846–1.112)	0.691
Prurigo (EC91.Z)	296 (8.2%)	174 (7.5%)	470 (7.9%)	0.914 (0.763–1.094)	0.350
Scabies (1G04)	222 (6.1%)	231 (9.9%)	453 (7.6%)	1.617 (1.355–1.930)	**<0.001**
Dermatophyte infections (1F28.Z)	159 (4.4%)	95 (4.1%)	254 (4.3%)	0.929 (0.724–1.191)	0.599
Lichen simplex (EA83)	99 (2.7%)	100 (4.3%)	199 (3.3%)	1.570 (1.195–2.062)	**<0.001**
Urticaria (EB05)	126 (3.5%)	70 (3.0%)	196 (3.3%)	0.863 (0.643–1.151)	0.334
Pityriasis versicolour (1F2D.0)	121 (3.3%)	69 (3.0%)	190 (3.2%)	0.886 (0.662–1.186)	0.450
Pityriasis Rosea (EA10)	113 (3.1%)	52 (2.2%)	165 (2.8%)	0.715 (0.517–0.989)	**0.043**
Folliculitis/pseudofolliculi tis (ED9Y)	90 (2.5%)	64 (2.7%)	154 (2.6%)	1.105 (0.806–1.516)	0.559

*Note*: A *p*‐value <0.05 was considered significant.

**TABLE 4:** Multivariate analysis of prevalent dermatoses in the general population

Skin disease (ICD‐11 CODE)	Pre‐COVID‐19 era (*n* = 3619)	COVID‐19 era (*n* = 2604)	Total	aOR (95% CI)	*p*‐value
Scabies (1G04)	222 (6.1%)	231 (9.9%)	453 (7.6%)	1.640 (1.235–2.177)	**0.001S**
Lichen simplex (EA83)	99 (2.7%)	100 (4.3%)	199 (3.3%)	0.753 (0.539–1.051)	0.095 NS
Pityriasis Rosea (EA10)	113 (3.1%)	52 (2.2%)	165 (2.8%)	1.697 (1.400–2.057)	**<0.001S**

*Note*: A *p*‐value <0.05 was considered significant.

**FIGURE 1:**
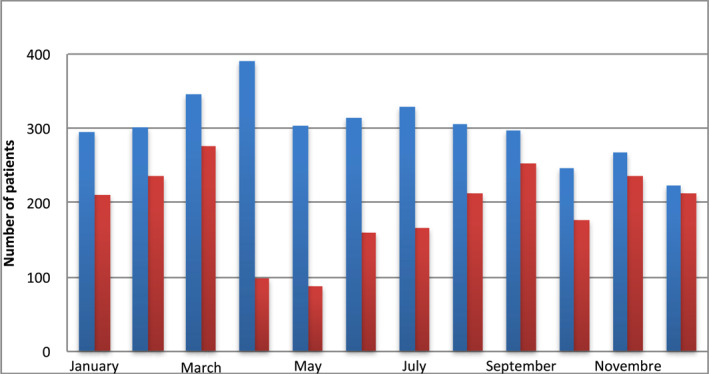
Variations in consultations over the months before and after COVID‐19's first reported case in Cameroon

There was a 35.6% drop in consultation frequency during the COVID‐19 era as denoted in Figure 1. This decrease was most significant in April and May 2020 where less than a third of cases of the previous year were consulted. Amongst patients aged >50 years, there was no difference in the consultation frequency before COVID‐19 (13.2%, *n* = 479/3618) and after COVID‐19 (13.7%, *n* = 321/2328), [OR = 1.492 (1.362–1.632) 95% CI, *p* = 0.958]. The most common cause for consultation was eczema (18.0%). There was a sharp increase (+5.8%) in the prevalence of eczema [OR = 1.373 (1.022–1.844) 95% CI, *p* = 0.039], scabies (+5.7%) [OR = 2.238 (1.330–3.767) 95% CI, *p* = 0.003] in the COVID‐19 era as shown in Table 5. On multivariate analysis of prevalent dermatoses in patients above 50 years, scabies [OR = 2.616 (1.488–4.601) 95% CI, *p* = 0.001] and eczema [OR = 1.605 (1.112–2.316) 95% CI, *p* = 0.011] showed a significant increase in consultation frequency in the COVID‐19 era as shown in Table 6.

**TABLE 5:** Top diagnoses amongst elderly patients (>50 years) considered risk group for COVID mortality

Skin disease (ICD‐11 CODE)	Pre‐COVID‐19 era (*n* = 479)	COVID‐19 era (*n* = 321)	Total (*n* = 826)	OR (95% CI)	*p*‐value
Eczema (EA80)	75 (15.7%)	69 (21.5%)	144 (18.0%)	1.373 (1.022–1.844)	**0.039**
Acne (ED80.Z)	11 (2.3%)	5 (1.6%)	16 (2.0%)	0.678 (0.238–1.934)	0.609
Prurigo (EC91.Z)	17 (3.5%)	6 (1.7%)	23 (2.9%)	0.527 (0.210–1.321)	0.198
Scabies (1G04)	22 (4.6%)	33 (10.3%)	55 (6.9%)	2.238 (1.330–3.767)	**0.003**
Pityriasis versicolour (1F2D.0)	9 (1.9%)	1 (0.3%)	10 (1.3%)	0.166 (0.021–1.302)	0.057
Dermatophyte infections (1F28.Z)	26 (5.4%)	21 (6.5%)	47 (5.9%)	1.205 (0.690–2.105)	0.541
Lichen simplex (EA83)	21 (4.4%)	15 (4.7%)	36 (4.5%)	1.066 (0.558–2.036)	0.863
Urticaria (EB05)	9 (1.9%)	9 (2.8%)	18 (2.3%)	1.492 (0.599–3.718)	0.467
Pityriasis rosea (EA10)	4 (0.8%)	0 (0%)	4 (1.9%)	NA	NA
Folliculitis/pseudofolliculitis (ED9Y)	7 (1.5%)	8 (2.5%)	15 (1.9%)	1.705 (0.625–4.657)	0.301

*Note*: A *p*‐value <0.05 was considered significant.

**TABLE 6:** Multivariate analyses of prevalent dermatoses amongst patients above 50 years

Skin disease (ICD‐11 CODE)	Pre‐COVID‐19 era (*n* = 479)	COVID‐19 era (*n* = 347)	Total (*n* = 826)	OR (95% CI)	*p*‐value
Eczema (EA80)	75 (15.7%)	69 (21.5%)	144 (18.0%)	1.605 (1.112–2.316)	**0.011**
Scabies (1G04)	22 (4.6%)	33 (10.3%)	55 (6.9%)	2.616 (1.488–4.601)	**0.001**

*Note*: A *p*‐value <0.05 was considered significant.

## 5 DISCUSSION

This research aimed at describing the epidemiology of skin diseases at the outpatient dermatology clinic in a tertiary hospital in Cameroon. We also intended to describe the variations of these consultations since COVID‐19 was reported in Cameroon. This research is expected to help health policy makers in understanding the burden of skin diseases and guide training sessions of health professionals in order to improve dermatology care in our setting.

The mean age, in our overall sample population, was 28.9 (±18.0) years, with a median age of 27.0 (IIQ 23) years and an age range of 1 month–89 years. When analysed as groups, patients aged 18–40 years, that is, young adults (49.4%) and patients aged 0–17 years, that is, paediatric group (26.2%) predominated. This shows that three‐fourths patients consulting at the dermatology clinic are youths (≤40 years). This is in accordance with other studies carried out in West Africa: Ghana and Nigeria.^12^, ^13^, ^14^ Contrarily, in western countries, the mean age and predominant age groups are generally older, above 50 years.^15^, ^16^ In a study published in 2009 in Cameroon, age range from few weeks to more than 80 years have been similarly described.^5^, ^17^ This reinforces the fact that skin diseases affect all age groups. Dermatology outpatient consultations have always been considered to be predominated by females^6^, ^18^ as confirmed by the current study. However many other studies in Africa, the United States and the Europe report a male to female ratio always around 1:1.^5^, ^13^, ^15^ We can therefore conclude that at Laquintinie, females utilise dermatology outpatient services more than men. This is probably because females pay more attention to details concerning their appearance than men and are therefore more likely to seek care. In our study, most patients were students: primary, secondary and university. They constituted the bulk of the population (40.3%). This is in accordance with the most prevalent age groups in the sample population. This trend has not been described in other studies in Cameroon. It was rather found that, the agricultural sector and workers of the industrial sectors take the lead.^19^, ^20^

Most patients consulting at dermatology unit were inhabitants of the economical capital city, Douala. Only 5.6% of patients lived out of Douala. However, most patients came from areas (subdivisions) away from the subdivision in which the hospital is located (Douala I). Most patients came from Douala III (1 020 000 inhabitants) and Douala V (859 988 inhabitants). These areas are the most populated areas of Douala (3 793 000 inhabitants). Douala III and V combined constitute about 50% of the population of Douala^21^ and this could explain why most patients come from these two areas. We think one major reason could be financial. Most private clinics that offer expert dermatology services are located in Douala 1 and have a consultation fee that ranges from 10.000 to 25.000 XAF (18–45 USD). Laquintinie as a second category hospital offers expert level dermatology services at a consultation fee of 3000 XAF (5 USD). The only alternative public centre offering expert dermatology services is located in Douala V and does so at a consultation fee of 7000 XAF (12 USD). No public hospital offers expert dermatology services in Douala III.

Skin infections and infestations accounted for 30.3% of cases; dermatitis and eczematous conditions, acne and acneiform disorders, papulosquamous disorders, and skin tumours were observed in 19.4%, 14.3%, 11.7% and 10.3% of cases, respectively. This trend has been described in other community‐based African studies, where, infectious dermatoses predominate the clinical picture.^13^, ^14^, ^16^, ^22^, ^23^ This trend is consistent in Africa, though some studies denote allergic/hypersensitivity conditions as being the most frequent dermatoses (30.4%),^6^, ^12^ a pattern also reported in some western countries.^24^, ^25^ Nonetheless, malignant and pre‐malignant conditions have also been reported as the most common cause of consultation in some western studies.^4^

In the current study, acne was found to be the single most frequent condition in our setting. This trend can be explained by the fact that most patients were youths, an age group where the condition predominates. Nonetheless, this trend has been described recently in some studies even in Africa.^14^ Acne is the single most condition leading to altered quality of life and thus patients with this condition tend to seek care and adhere to treatment more than patients with other skin conditions.^26^ The second commonest skin disorder in our setting was eczema as similarly reported in Nigeria^12^ and Cameroon.^17^ Scabies was the fourth most prevalent single dermatosis. It is still prevalent in our context and in other developing countries. One of the highest prevalence has been described in Fiji, in welfare houses (31%).^27^ In 2017, the WHO classified scabies and other ectoparasites as neglected tropical diseases.^28^ Its high prevalence in our context may be due to the high promiscuity still occurring in our setting. Measures to eradicate or reduce prevalence of this illness are important to set in place since this illness is highly preventable.^29^ The increase in prevalence of scabies can be explained by the fact that many people could have stayed indoors hence increasing the transmissibility of the infestation. The high prevalence of eczema in the COVID‐19 era can make us suspect lesions associated with COVID‐19 as has been described in literature.^30^ Moreover, the routine practice of measures to prevent COVID‐19 such as frequent hand washing and frequent wearing of masks could be incriminated in the rise of eczema in the COVID‐19 era.^28^, ^31^

Elderly patients (>50 years), who were considered the most vulnerable population, did not witness a significant change in consultation frequency. There was however a sharp increase in the proportion of elderly patients consulting for eczema. This suggests that eczema is a highly debilitating condition that prompted risk taking (i.e., going outdoors despite the associated risks) in this elderly population. The second skin condition that prompted consultations amongst this vulnerable group was scabies. This goes to conclude, like studies had done before,^32^, ^33^ that eczema and scabies, being highly pruritic conditions, are debilitating enough to be considered conditions needing emergency care.

Challenges to access dermatology services have been extensively described in literature. The pandemic has reduced physician to patient contacts. Less patients seek care for fear of contracting COVID‐19. After Laquintinie was declared as an exclusive COVID‐19 treatment centre (April 2020), the consultation rate drastically reduced. There was a 35.6% decrease in consultation frequency in the COVID‐19 era. When tailoring the drop in dermatology consultations to overall hospital consultations, we realised that the COVID‐19 era witnessed a drop of 1.1% (*p* < 0.001) of overall consultations as a consequence of the drop in dermatology consultations. We could therefore conclude that the COVID‐19 era witnessed a real drop in consultation frequency independent of fluctuations of hospital's overall consultations. In a bid to prevent such a scenario in case of worsening of the pandemic or a new pandemic with similar transmissibility, tele‐dermatology has been suggested as an adequate solution.^34^ A recent study^35^ reported that up to 72.8% of dermatology consultations might not require face‐to‐face examination and could be safely managed through tele‐dermatology. Though not practised officially on large scale, teledermatology services have been piloted before in Cameroon.^36^ More feasibility studies on the implementation of tele‐dermatology clinics in Cameroon and Africa in general should be carried out in order to curb the barriers to access to dermatology services especially during pandemics.

This research work has some limitations. Firstly, this research was retrospective and as such much data could not be fully retrieved from patients. Moreover, this research was carried out in a single centre (secondary level facility) in a single town in Cameroon. Generalisation of these findings to the whole town or even the whole country is impossible for these reasons. Nonetheless, one of the strengths of this research lies in the fact that it’s a novel research as it addresses consultation challenges in the COVID‐19 era and serves as a pioneer research for studies with more robust methodologies. Furthermore, this research analysed data from more than 5000 dermatology consultations in a Sub‐Saharan country. This sample is substantial, and though not perfect, can serve as a reference for further studies on dermatoses in Cameroon.

## 6 CONCLUSION

We therefore conclude that overall, infectious dermatoses predominate at outpatient dermatology consultations. However, the single most prevalent dermatology condition is Acne. Three in four patients consulting at dermatology clinic are below 40 years, with a female predominance. Consultation frequency decreased in the COVID‐19 era but trends in consultations were rather unaltered. Patients above 50 years consulted more frequently for scabies and eczema in the COVID‐19 era. More studies should be conducted to understand these trends better at a national level and envisage training of health personnel on prevalent dermatoses.

## CONFLICT OF INTEREST

The authors declare no conflict of interest.

## AUTHOR CONTRIBUTIONS

**Edgar Mandeng Ma Linwa:** Conceptualization; Data curation; Formal analysis; Writing – original draft; Writing – review & editing. **Odette Berline Sigha:** Data curation; Writing – review & editing. **Angelique Jacquie Djeumen Touka:** Data curation; Writing – review & editing. **Charlotte Eposse Ekoube:** Writing – review & editing. **Esther Eleonore Ngo Linwa:** Writing – review & editing. **Michael Ngenge Budzi:** Writing – review & editing. **Martin Geh Meh:** Writing – review & editing. **Henry Fomukong Nzozone:** Writing – review & editing. **Emmanuel Armand Kouotou:** Writing – review & editing. **Anne‐Cecile Bissek:** Writing – review & editing.

## Data Availability

Raw data were generated at Laquintinie Hospital Douala. Derived data supporting the findings of this study are available from the corresponding author Dr. Edgar Mandeng Ma Linwa on request.
